# Recycled algae-based carbon materials as electroconductive 3D printed skeletal muscle tissue engineering scaffolds

**DOI:** 10.1007/s10856-021-06534-6

**Published:** 2021-06-21

**Authors:** Selva Bilge, Emre Ergene, Ebru Talak, Seyda Gokyer, Yusuf Osman Donar, Ali Sınağ, Pinar Yilgor Huri

**Affiliations:** 1 Department of Chemistry, Ankara University Faculty of Science, Ankara, Turkey; 2 Department of Biomedical Engineering, Ankara University Faculty of Engineering, Ankara, Turkey; 3 Ankara University Biotechnology Institute, Ankara, Turkey

## Abstract

Skeletal muscle is an electrically and mechanically active tissue that contains highly oriented, densely packed myofibrils. The tissue has self-regeneration capacity upon injury, which is limited in the cases of volumetric muscle loss. Several regenerative therapies have been developed in order to enhance this capacity, as well as to structurally and mechanically support the defect site during regeneration. Among them, biomimetic approaches that recapitulate the native microenvironment of the tissue in terms of parallel-aligned structure and biophysical signals were shown to be effective. In this study, we have developed 3D printed aligned and electrically active scaffolds in which the electrical conductivity was provided by carbonaceous material (CM) derived from algae-based biomass. The synthesis of this conductive and functional CM consisted of eco-friendly synthesis procedure such as pre-carbonization and multi-walled carbon nanotube (MWCNT) catalysis. CM obtained from biomass via hydrothermal carbonization (CM-03) and its ash form (CM-03K) were doped within poly(ɛ-caprolactone) (PCL) matrix and 3D printed to form scaffolds with aligned fibers for structural biomimicry. Scaffolds were seeded with C2C12 mouse myoblasts and subjected to electrical stimulation during the in vitro culture. Enhanced myotube formation was observed in electroactive groups compared to their non-conductive counterparts and it was observed that myotube formation and myotube maturity were significantly increased for CM-03 group after electrical stimulation. The results have therefore showed that the CM obtained from macroalgae biomass is a promising novel source for the production of the electrically conductive scaffolds for skeletal muscle tissue engineering.

## Introduction

Tissue engineering is a multidisciplinary research field that aims to produce functional tissues and organs to regenerate or to replace the malfunctioning ones. In order to develop functional outcomes, cells as appropriate to the tissue type, biomaterials mimicking the native extracellular matrix, as well as bioactive agents to induce the activity of the grafted and host cells are used. Although most of the tissues possess robust self-regeneration capacity upon injury, there is a volumetric limit above which the defect needs to be structurally and mechanically supported in order to obtain functional regeneration [1]. For example, skeletal muscle regenerates itself in response to injury to maintain its function. The satellite cells that are the quiescent native progenitor cells of the tissue are activated in the case of injury, and the cascade of proliferation and differentiation into multinucleated myofibers initiates [2]. However, volumetric muscle loss that is caused by reasons including traumatic injuries or tumor removal cannot be fully repaired by this self-repair mechanism. Current clinical treatments including the use of autologous muscle flaps, use of allografts or other filler materials have their own limitations. Therefore, tissue engineering is seen as a promising approach in the production of viable functional tissues and organs including the skeletal muscle [3–5].

Skeletal muscle constitutes nearly 45% of the total body mass and enables voluntary movement of the skeleton by contraction [1]. The architecture of the tissue consists of parallel-aligned muscle fibers that are packed together. These packed fibers are made out of myofibril clusters that are responsible for the contraction of the muscle [6, 7]. The microarchitecture and physiology of the native tissue gives clues about how the engineered skeletal muscle construct should be.

Scaffolds play critical roles in directing the cell fate, which necessitates the precise control of the scaffold microarchitecture [8, 9]. 3D printing has increasing importance in tissue engineering strategies as it enables the production of desired shapes with a layer by layer approach, even when they have high structural complexity [10]. Scaffolds with complex structures can be designed and produced precisely with this technique using various biomaterials according to the computer-aided design (CAD) [11–15]. Fused deposition modeling (FDM) is a frequently applied technique for 3D printing, in which biomaterials are either dissolved within the corresponding solvents, or used in molten form that are extruded in the form of filaments. These filaments are then stabilized for layer by layer fabrication of 3D scaffolds for various tissues including cartilage, bone, cornea and for neural and cardiac tissue engineering [16–20] as well as skeletal muscle [21–24]. The parallel-aligned structure of the scaffold is an important parameter for structural biomimicry in the case of the latter [25, 26].

The central nervous system produces electrical impulses which leads to voluntary muscle contraction. These impulses must be generated with an external source in the in vitro applications, while using electrically conductive scaffolds for enhanced regenerative outcomes. For example, it was shown that the electrical stimulation of cells seeded on conductive scaffolds composed of polyurethane-carbon nanotubes (CNT), increase adhesion and differentiation of C2C12 cells and induce the formation of myotubes [27]. Specifically, it was reported that the electrical stimulation leads to the enhancement of myofiber production [28, 29]. Along with its importance in the skeletal muscle tissue engineering, the use of biomimetic electrical stimulation also finds important applications in the engineering of functional cardiac [30, 31] and nervous [32, 33] tissues. Therefore, in addition to structural biomimicry by 3D alignment, mimicking the electroactive nature of the skeletal muscle tissue by incorporation of electroconductive agents is an important strategy to enhance the adhesion, proliferation, and orientation of the cells [25, 34, 35]. For example, myotube formation was enhanced when C2C12 seeded aligned fibers were stimulated by an electrical field [23]. Chen et al. [34] used C2C12 myoblasts to investigate the orientation and myotube formation on aligned and random PCL scaffolds in the presence and absence of polyaniline (PANI) as the conductive material. Results represented the electrically conductive PCL/PANI scaffold with aligned structure enhances the myotube formation and maturation. Also, the electrically conductive and aligned collagen/polypyrrole (PPy) scaffold enhanced myoblast orientation and induced myotube formation [26]. The added effect of the combination of electrical stimulation together with an aligned structure on the myogenic differentiation and regenerative capacity was shown in other studies, as well [35, 36].

Application of electrical impulse with various amplitudes, width and frequency have been studied to stimulate the muscle cells [37]. It was indicated that high electrical pulse can disrupt the cells and the scaffold, whereas a low amount of electrical pulse can be ineffective [38]. For example, myoblasts were exposed to 2 ms pulses at 12 V, with a frequency of 1 Hz by Tarum et al. [39]. It was reported that myoblasts were differentiated into multinucleated myotubes when stimulated, and a significant increase in the size of the mature myoblasts along with an enhanced level of muscle specific marker expression was obtained [39]. Park et al. investigated the effects of electrical stimulation on the C2C12 seeded collagen scaffolds [29]. Different electrical voltage and stimulation frequency values were evaluated in terms of myogenic differentiation. The values 1 Hz/5 V- and 2 Hz/5 V were determined as the appropriate values for stimulation [29]. It was reported that stimulation in the range of 65–200 mV/mm was effective to differentiate induced pluripotent stem cells into cardiomyocytes [40]. Therefore, electrical stimulation regime needs to be finetuned according to the target tissue, cell type and the biomaterial used [41].

There are several approaches in the literature that use conductive materials towards skeletal muscle repair. Among these, graphene oxide and other carbon-based materials were recently used in combination with polymers to induce myogenic differentiation [42–46]. For example, the presence of nano-graphene oxide within polyurethane matrix enhanced the expression of myogenic genes when investigated with real-time PCR [42]. The CNT scaffold induced myoblast differentiation and the use of aligned CNTs further enhanced the formation of multinucleated myotubes when compared to carbon foam scaffolds [44]. Also, gold nanoparticles [47], PANI [34], and PPy [48] have been utilized as conductive agents. However, these materials are very costly, moreover, their synthesis generally consist of toxic and environmentally hazardous chemicals [49]. In order to overcome these, we have studied the potential of conductive CM synthesized by means of the eco-friendly hydrothermal carbonization (HTC) method as alternative conductive fillers for skeletal muscle tissue engineering scaffolds.

HTC is a biomass conversion technique applied under mild temperature (180–260 °C) and pressure (generally autogeneous) conditions [50]. In the HTC process, biomass turns into hydrochar as a result of various reactions such as dehydration, decarbonylation, decarboxylation, polymerization, poly-condensation, aromatization, and re-condensation [51]. The main advantage of HTC over other thermo-chemical conversion techniques (such as pyrolysis, gasification, and incineration) is its ability to convert wet raw and waste material into a solid CM (hydrocarbon, char) with relatively high yields, functional group and electrical conductivity without pre-dewatering and drying [52]. Valuable specifications of these CM such as high porosity and conductivity are suitable for the adsorption of large molecules and electrochemical signal transduction, respectively [53].

In recent years, many waste-based biomass sources such as sewage sludge [54], hazelnut shells [55], tea leaves [56] etc. are used in the HTC process. In this study, *Cystoseira* macroalgae was used as a source of biomass in order to obtain the filler material with electrical conductivity [50]. Algae are among the most demanded biomass sources [57, 58]. Macroalgae, specifically, contain a wide range of metals due to its natural habitat of “salty seawater” compared to other microalgae forms [59]. This nature of macroalgae gives it such an important advantage as electrical conductivity (42–53 mS cm^−1^) [60]. In this study, the HTC method was applied twice (CM-01, CM-02), and the degree of graphitization was further increased by multi-walled CNT catalysis (CM-03) in order to enhance the electrical conductivity. The ash form CM-03K was also used in order to better show the effect of the material to provide a conductive matrix.

In this study, the aim was to prepare 3D printed electroconductive skeletal muscle tissue engineering scaffolds using conductive materials obtained from natural sources. Applications of such recycled biomass-based conductive materials as fillers for tissue engineering scaffolds has not been performed up to date, to the best of our knowledge. The CM-03, and its ash form CM-03K were doped in PCL and 3D printed with a parallel-aligned pattern. C2C12 mouse myoblasts were seeded on these conductive scaffolds, and electrical stimulation was applied with the purpose of enhancing myotube formation. The potential of the novel conductive fillers for use in tissue engineering was investigated in the in vitro settings.

## Materials and methods

### Materials

PCL (average MN 80,000) was obtained from Sigma Aldrich (USA). Dichloromethane (DCM) was purchased from Merck-Millipore (USA). C2C12 mouse myoblast cell line (CRL-1772™) was obtained from ATCC^®^ (UK). Dulbecco’s Modified Eagle Medium (DMEM, high and low glucose), fetal bovine serum (FBS), donor horse serum (DHS), and penicillin-streptomycin (P/S) were purchased from Biological Industries (Israel). Bovine serum albumin (BSA) was obtained from Sigma (USA). Alamar Blue cell proliferation assay was obtained from Thermo Fisher (USA). Immunofluorescence staining was performed by using primary antibodies against myogenin (ab1835) and myosin heavy chain, obtained from Abcam (UK). Secondary antibodies goat anti-mouse (Alexa Fluor^®^ 488) (ab150113) and goat anti-rabbit (Alexa Fluor^®^ 594) (ab150080) were purchased from Abcam (UK). Phalloidin (Alexa Fluor^®^ 488) and DAPI were obtained from Invitrogen (USA).

The *Cystoseira* algae species used in the preparation of CM-03 were collected from the Marmara Sea (Turkey). MWCNT used for catalysis was purchased from Sigma Aldrich.

### Synthesis of the recycled conductive material

*Cystoseira* algae was completely dried at room temperature and was mechanically grounded and sieved to 150 μm mesh. Pre-carbonization (usually applied at low temperature) process was carried out during the synthesis of CM-03. For this, 20 g of algae biomass was mixed with 400 mL of distilled water and then carbonized at 180 °C for 2 h.

The CM obtained from the pre-carbonization procedure (CM-01) was re-subjected to the HTC conditions. For this, 3 g of CM-01 was dispersed in 200 mL of distilled water and the mixture was carbonized at 250 °C for 8 h. The product was labeled as CM-02.

MWCNT was used as a catalyst in the second HTC run, in order to improve the electrical conductivity and to increase the degree of graphitization. Briefly, 3 g of CM-01 and 0.03 g of MWCNT were dispersed in 200 mL of distilled water and the mixture was carbonized at 250 °C for 8 h in order to obtain CM-03.

### Characterization of the recycled conductive material

A series of analyses were carried out to determine the structure of the CMs synthesized. Scanning electron microscopy (SEM) (QUANTA 400 F Field Emission) analysis was applied to determine the surface morphology and size of the CMs. Electrical conductivity properties were studied with X-Ray Fluorescence (XRF) and Raman Spectroscopy analysis. The concentration of each metal element in the carbon material was determined using the polarized energy-dispersive XRF technique. Four grams of powdered CM was mixed with 0.9 g of wax and the mixture was pressed at 15 N in an automatic press machine to obtain thick pellets with diameter of 32 mm. XRF analysis was performed by a Spectro XLAB 2000 PEDXRF spectrometer equipped with Rh anode X-ray tube. Raman Spectroscopy analysis was carried with a Bruker SENTERRA Dispersive Raman spectrometer, which is equipped with an Olympus confocal microscope mounted onto a crane. X-Ray Photoelectron Spectroscopy (XPS) analysis (PHI‐5000 Versa probe using an Al monochromatic X‐ray anode) was also applied to determine the structure and functional groups of the CMs. Elemental analysis was applied to determine the basic elements forming the structure of CMs. Elemental analysis were carried out by the Eurovector 3018 CHNS analyzer. HTC technique was performed in an autoclave system (Parr Instrument) with 300 mL internal volume. The autoclave system consists of a temperature-pressure control unit and a leakproof vessel.

### 3D printing of the scaffolds

Extrusion-based 3D printing system (Bioplotter Starter Series, Envisiontec) was used to prepare the scaffolds. For this purpose, PCL was dissolved in DCM (70% w/v), and the synthesized CMs (CM-03 and CM-03K) were dispersed at 2% (w/v) within the solution. The low-temperature deposition of filaments was carried out with 25-gauge needle tip, the printing pressure was 1.4–2.0 bar, and the extrusion speed was 4.0–5.0 mm/s speed. The scaffolds were prepared in parallel aligned fibers for mimicking the natural skeletal muscle architecture. The size of the printed scaffolds were 15 mm × 5 mm × 0.5 mm and the distance between two strands of the scaffold was 0.8 mm. Scaffolds fabricated from undoped PCL was used as the control group.

Mechanical properties of the scaffolds were determined by uniaxial tensile testing using a universal testing machine (Shimadzu AGS-X) since the major force exerted on the majority of skeletal muscles is the continuous uniaxial cyclic stretch under physiological conditions. Tensile testing was applied on samples with dimensions of length: 15 mm, width: 5 mm and thickness: 0.5 mm (*n* = 5) and samples were elongated under 5 kN load at a speed of 1 mm/min. The applied load and strain of the samples are recorded as a function of time. Ultimate tensile strength (UTS), % elongation at break (EAB) and Young’s Modulus (E) values of PCL, CM-03/PCL and CM03K/PCL scaffolds were calculated.

### Cell Seeding, culture, and electrical stimulation

**Fig. 1:**
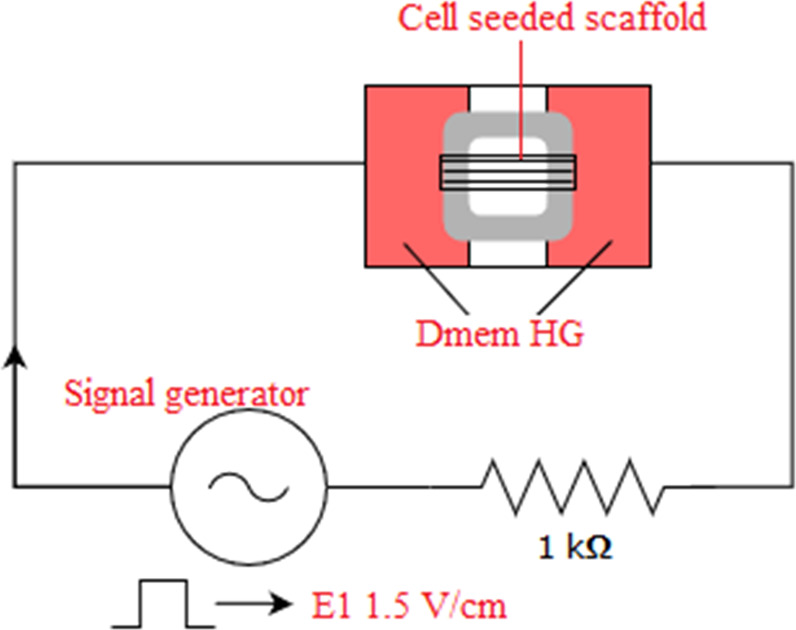
Schematic representation of the electrical stimulation system

C2C12 cells were expanded under standard culture conditions within high glucose DMEM supplemented with 10% FBS and 1% P/S. Sub-confluent cells were detached from the flasks and were seeded at P5 on 3D printed scaffolds at 100,000 cells/sample. All 3D printed scaffolds were sterilized by soaking within 70% ethanol prior to cell seeding. Following overnight incubation after cell seeding, differentiation medium (low glucose DMEM with 10% DHS and 1% P/S) was applied for 7 days. Beginning from day 3, E1 sub-groups of all scaffolds were exposed electrical stimulation with 1.5 V/cm rectangular pulse sequence of 1 ms duration and 1 Hz frequency 5 min/day in the laminar flow cabinet (Fig. 1). E0 sub-groups did not receive electrical stimulation.

### Cell viability, morphology, and immunocytochemistry

Viable cell number was assessed with Alamar Blue cell proliferation assay at 1, 4, and 7 days of culture. Briefly, Alamar Blue solution (10%, 1 mL) in colorless DMEM was added to the wells after washing with phosphate buffered saline solution (PBS) and incubated at 37 °C and 5% CO_2_ for 1 h. A total of 200 μL of the test solution was transferred to a 96-well plate and absorbance was determined at 570 and 595 nm using the plate reader at the end of the incubation period. The test medium in the wells was then discarded, rinsed with PBS, fresh culture media was added and the incubation was continued within the wells. The percent reduction of the Alamar Blue dye was correlated to viable cell number through a calibration curve.

Cell morphology on the scaffolds was studied by SEM (Leica Cambridge S360) analysis on day 7. For that, samples were fixed with paraformaldehyde (3.7% in piperazine-N,N′-bis(2-ethanesulfonic acid) (PIPES) buffer, pH 6.8) for 1 h at RT. They were then washed with PIPES buffer several times and air-dried and sputter coated with gold prior to the SEM examination.

Investigation of the F-actin filaments within the cells was performed with immunostaining with Phalloidin. Briefly, cell seeded scaffolds were fixed with 3.7% paraformaldehyde for 30 min. After rinsing with PBS, 0.1% Triton X-100 was used to permeabilize the cell membranes. Samples were then incubated with 1% BSA for 30 min at 37 °C to prevent non-specific binding. Phalloidin (1:200) was incubated with the samples for 1 h at 37 °C. Cell nuclei were labeled with counterstaining with DAPI (1:1000) for 10 min at RT. All images were taken using a confocal laser scanning microscope (CLSM) (Leica TCS SPE). The figures were presented as vertical projections of 300 μm thick sections.

To determine the muscle-specific marker expression, immunofluorescent staining for muscle specific markers Myogenin and Myosin Heavy Chain was performed. Briefly, cell-seeded scaffolds were fixed, permeabilized and blocked against non-specific binding as described above. The samples were then incubated with primary antibodies mouse anti-myogenin (1:50) and rabbit anti-myosin heavy chain (1:100) diluted in 0.1% BSA at 4 °C overnight. Following this step, samples were incubated with secondary antibodies (Alexa Fluor^®^ 488-conjugated goat anti-mouse IgG (1:200) and Alexa Fluor^®^ 594-conjugated goat anti-Rabbit IgG (1:200)) for 1 h at room temperature. Nuclei were labeled using DAPI (1:1000). Images were taken using a CLSM. Myotube number and the maturation index were calculated from MHC staining. The maturation index was formed by proportioning myotubes containing five or more nuclei to total myotubes [61].

### Statistical analysis

All quantitative results were expressed as means ± standard deviation (*n* = 4). Data was analyzed with statistically significant values defined as *p* < 0.05 based on one-way analysis of variance (ANOVA) followed by Tukey’s test for determination of the significance of difference between different groups (*p* ≤ 0.05).

## Results and discussion

### Characterization of the recycled conductive material

#### Physicochemical properties

In this study, 3D printed electroconductive skeletal muscle tissue engineering scaffolds were prepared by using recycled CMs with natural conductivity. The natural conductivity of carbon materials is sourced by the metal content in their raw structures. Metal concentration content of raw and HTC treated forms of carbonaceous materials were determined by XRF analysis. Results of this analysis including the metal concentration values of *cystoseira* algae and CM-03 materials are presented in Table 1. According to these values, the high percentage of Si and Ca ions improve the natural electrical conductivity, since the high electron affinity metal ions (such as Ca, Si, Mg) have a positive effect on the electrically conductive structure. Therefore, the ash-containing part of the CM-03 sample (CM-03K) was also used in cell culture experiments. Elemental analysis and ash contents of the samples are summarized in Table 2. Results indicate that the degree of carbonization increases with the secondary HTC treatment.

**Table 1:** Metal concentration in cystoseria algae and CM-03

Elements (%)	Na	Mg	Al	Si	P	S	Cl	K	Ca	Fe
*Cystoseria* Algae	1.23	0.92	0.87	3.92	0.05	1.56	3.10	3.66	9.92	0.68
CM-03	0.04	2.19	1.54	6.15	0.13	1.72	0.05	0.55	19.34	1.40

**Table 2:** Ash content and results of elemental analysis of samples

Elements (%)	C	H	N	S	O	Ash
*Cystoseria* Algae	40.16	5.29	6.85	0.43	47.27	62.94
CM-01	41.56	4.49	1.65	1.17	51.09	40.05
CM-02	46.14	4.72	1.50	0.59	47.05	38.52
CM-03	48.19	4.21	1.55	0.51	45.54	36.95

3D CMs have better electrical and thermal conductivity than low-dimensional (0D, 1D, and 2D) carbonaceous materials since 3D carbon materials have a porous structure leading to a high surface/volume ratio. This property makes them perfect candidates for the electron transfer mechanism. In addition to the inherent conductivity, CMs can find potential application areas in biomedical research due to their extremely high mechanical strength and chemical stability.

The high surface/volume ratio obtained in CMs is very important in terms of electrical conductivity. In this study, the surface area of *Cystoseira* algae, and CM-01 samples could not be measured since they were below the detection limit. The surface areas of CM-02 and CM-03 materials were determined as 23.30 and 24.19 m^2^ g^−1^, respectively. The reason for the slightly higher surface area of CM-03 is considered to be due to the hydrophilicity gained due to the secondary HTC process [62].

#### Surface characterization

**Fig. 2:**
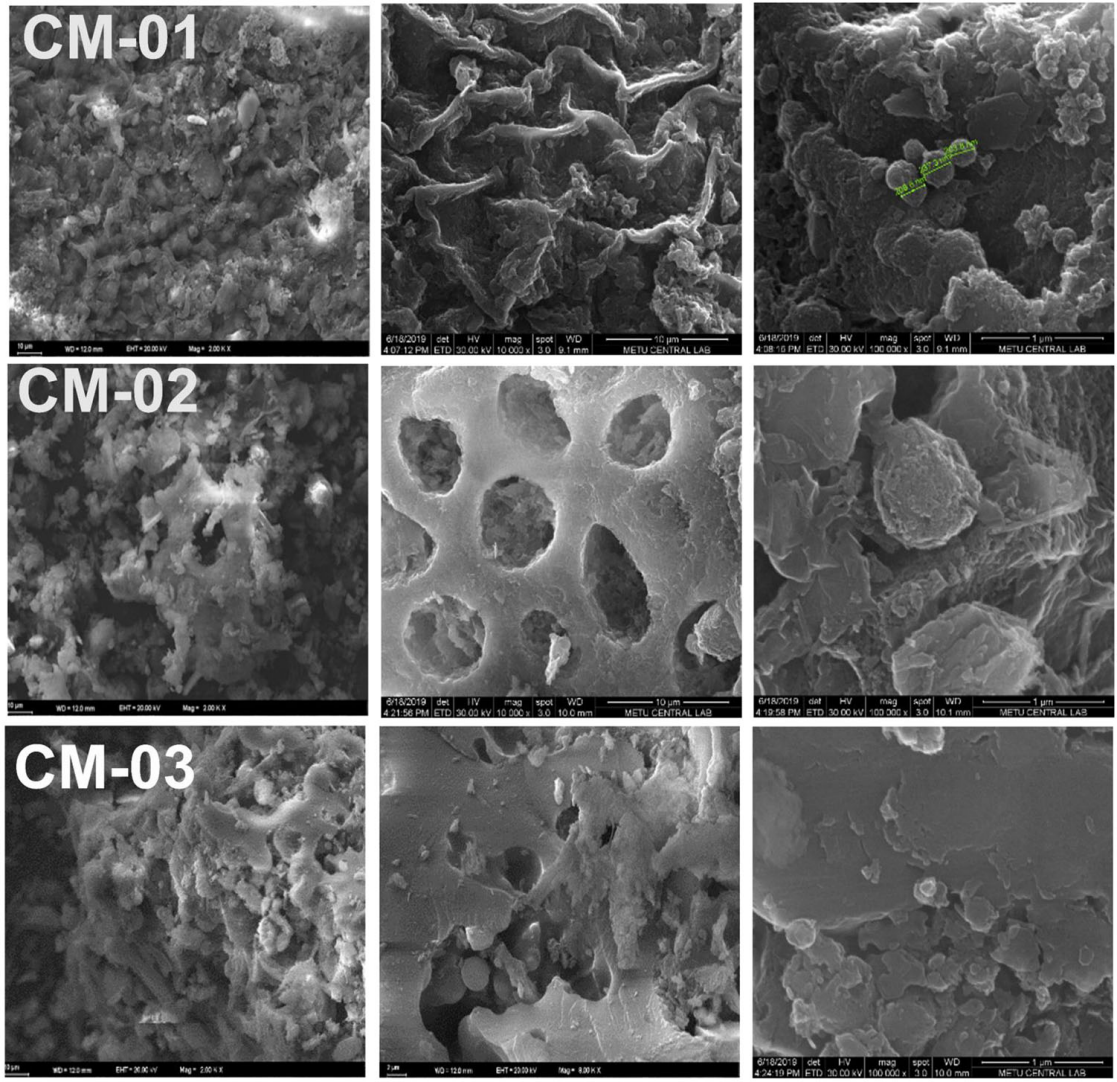
Surface morphology of CM-01, CM-02, and CM-03 samples. Scale bars indicate: 10 µm for the left and middle columns, 1 µm for the right column

Surface morphology of the synthesized materials were studied by SEM. It was observed that the surface morphology was changed pre- and post-HTC and after the second HTC process leading to the formation of hole-like structures in CM-02 (Fig. 2). This effect could be due to metal ions in the raw material.

#### Structural characterization

**Fig. 3:**
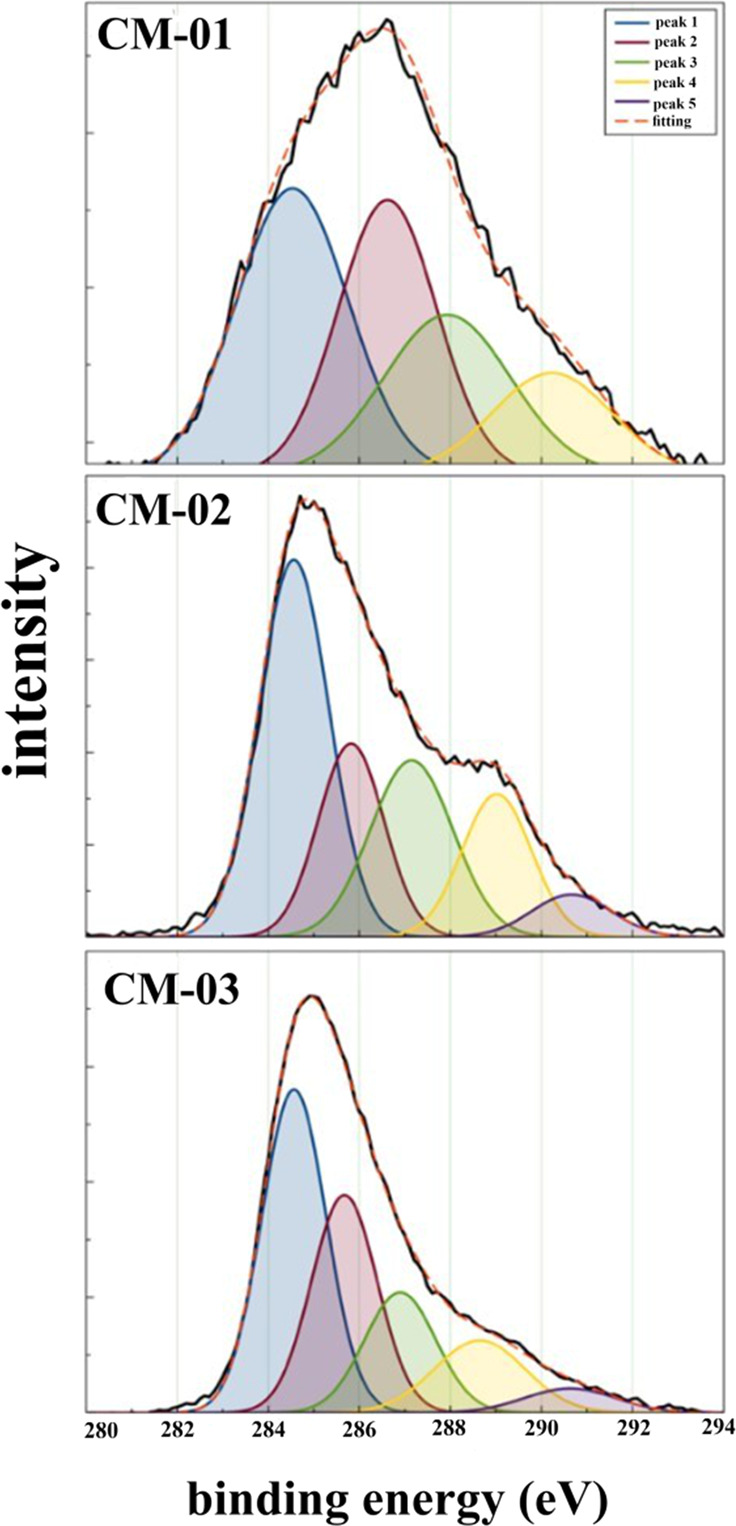
Typical spectra obtained by XPS analysis of CM-01, CM-02, and CM-03 samples

Various functional groups are formed on the material surface as a result of the aforementioned reactions during the HTC process. These groups were determined by XPS analysis, and inferences were made regarding the electrical conductivity of the materials. Figure 3 showed C1s core level spectra of char and nitrogen doped carbon sphere samples. According to spectra, five signals (284.6 eV (peak 1), 285.7 eV (peak 2), 287.3 eV (peak 3), 289.1 eV (peak 4), and 291.1 eV (peak 5)) were deconvoluted. These signals represent to carbon group (C = C, CHx, C–C), hydroxyl or ethers groups (-C-OR), carbonyl or quinone groups, and carboxylic groups, esters, or lactones (=COOR) and π-π transitions, respectively. When the XPS spectra were examined, π-π transitions (π-π transitions are one of the main parameters affecting the electron transfer of the material) could not be detected in CM-01. This may be due to the low temperature at (180 °C) pre-carbonization process [62].

**Fig. 4:**
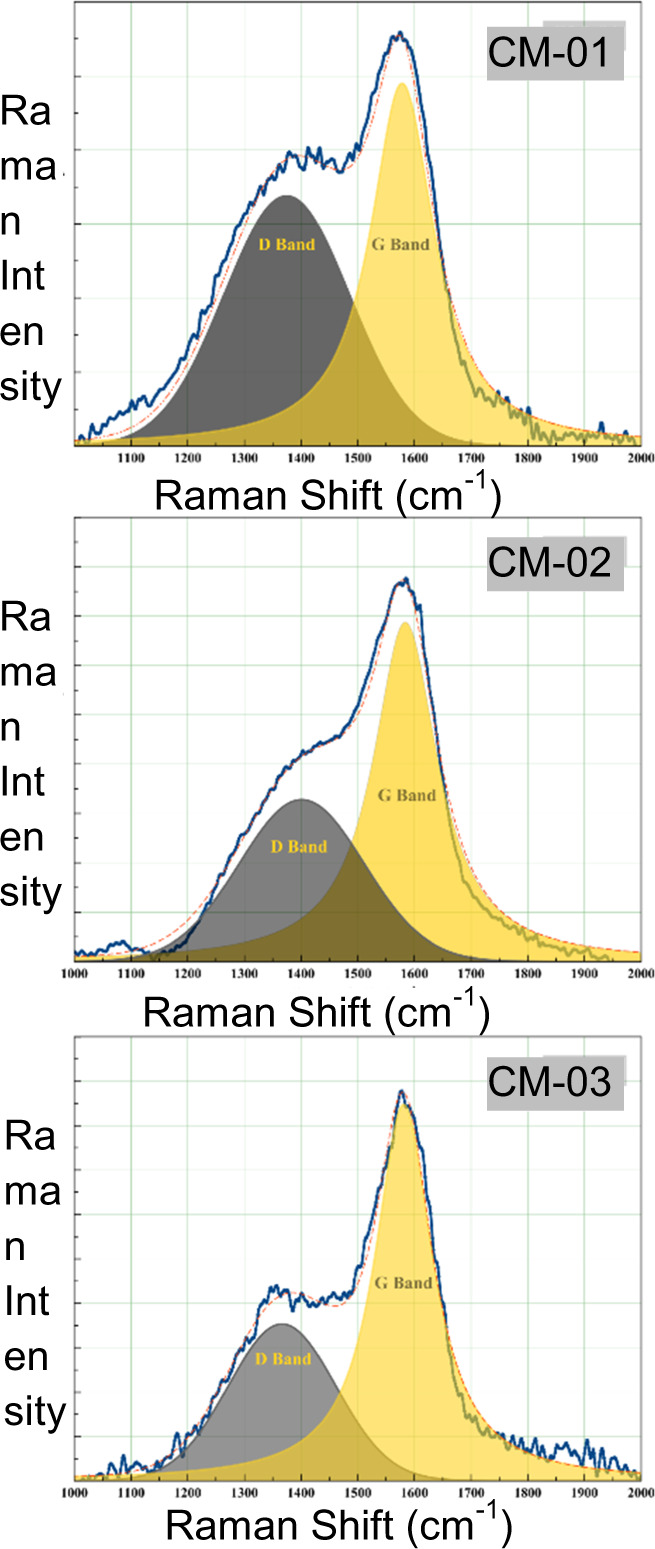
Raman spectra of CM samples

It is important to characterize the mechanical and electrical properties of the synthesized CMs in order to determine their applicability [63]. At this point, Raman analysis becomes a key factor in illuminating the structure of synthesized CMs since Raman scattering resonances are well-defined bands that can be obtained even if they are in minor amount in a matrix [64]. Raman analysis provides information about the graphitization degree of the synthesized CMs. Graphitization degree is a critical parameter that indicates the existence of electrical conductivity [65]. Figure 4 and Table 3 shows the results of the Raman analysis of the CM which characteristically have two bands. The first one corresponds to the D band, which is assigned to ring breathing vibrations in condensed benzene rings in partially hydrogenated amorphous carbon materials. The second band is generally called the G band and corresponds to the region of central vibration of carbon atom layer planes against each other. Analysis of results revealed that D and G bands were measured for all samples. The width of the ID and IG bands, and their ratio, allow calculation of the degree of graphitization for the samples (Fig. 4). The magnitude of this ratio is indicative of a lower degree of graphitization. In addition, the results support that the synthesized material has a carbonaceous skeleton and have electrical conductivity.

**Table 3:** Details of the results of Raman spectra

Sample ID	I_D_	I_G_	cm^−1^ (D band)	cm^−1^ (G band)	I_D_/I_G_
CM-01	16897	24510	1374	1578	0.69
CM-02	16403	34386	1401	1584	0.48
CM-03	3534	8478	1366	1581	0.42

### Characterization of 3D printed scaffolds

**Fig. 5:**
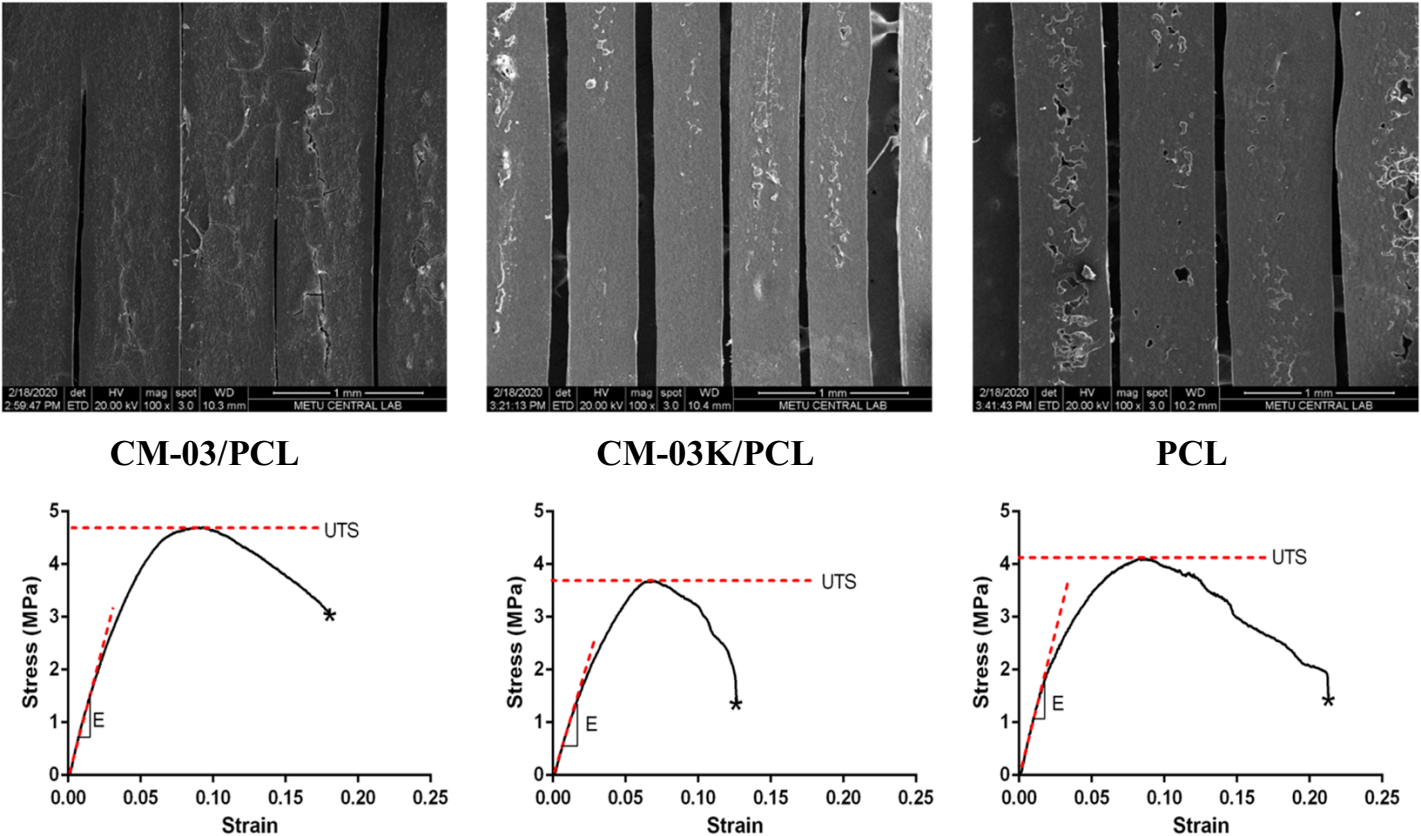
SEM observation (top row), and stress-strain curves (bottom row) for CM-03/PCL, CM-03K/PCL, and PCL scaffolds. Scale bars indicate: 1 mm

PCL and CM-03/CM-03K doped-PCL scaffolds were 3D printed with aligned fibrous structures. SEM observation revealed the production of non-porous structures when cut into half, and the fiber structures were not affected by the presence of the CMs with median D = 0.59 mm ± 0.14 mm (Fig. 5, top panel).

Representative stress-strain curves are presented in Fig. 5, bottom row, and the mechanical properties are given in Table 4. It was observed that no statistically significant differences were present due to the presence of conductive fillers, CM-03 and CM-03K, as compared to the bare PCL scaffolds. Myotube elongation is important for the normal function of natural skeletal muscle. It has been reported that the muscles are stretched at least 30% of their length during function (9). While CM-03K performs quite below 30%, PCL and CM-03 are relatively close to this value.

**Table 4:** Mechanical properties of CM-03/PCL, CM-03K/PCL, and PCL scaffolds (*n* = 5)

	Ultimate tensile strength UTS (MPa)	% Elongation at break	Young’s modulus E (MPa)
CM-03/PCL	4.69 ± 1.0	18.06 ± 5.5	98.80 ± 8.1
CM-03K/PCL	3.68 ± 0.7	12.66 ± 4.4	90.30 ± 5.6
PCL	4.10 ± 0.9	21.28 ± 4.2	104.50 ± 10.9

### Cell proliferation and morphology on the scaffolds

**Fig. 6:**
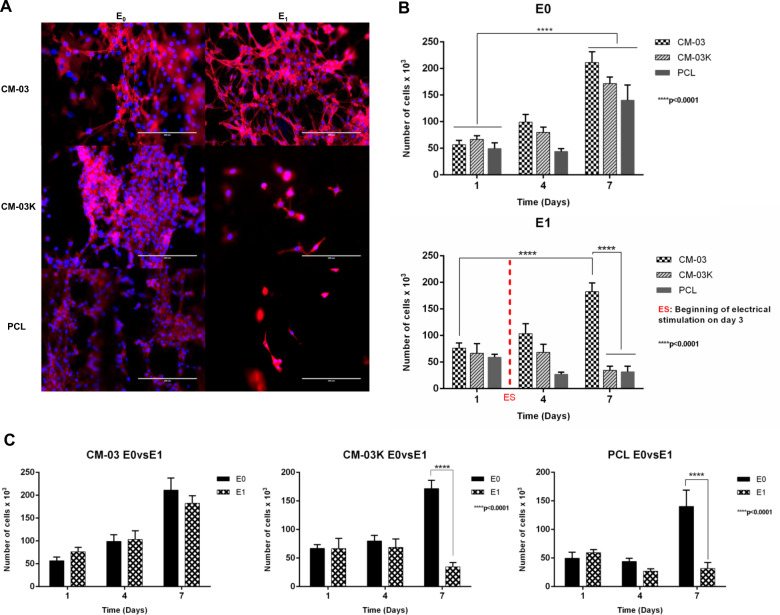
Phalloidin (Red)/DAPI (Blue) staining at day 7 (**A**). Alamar Blue cell viability results of E0 group without electrical stimulation and E1 group with 1.5 V electrical stimulation (**B**). Comparison of Alamar Blue cell viability results for each experimental group (**C**). Scale bar 200 µm

According to the cell viability test via Alamar Blue analysis, E0 sub-groups of all scaffolds have significantly more viable cells on day 7 compared to day 1. However, after electrical stimulation, only the CM-03/PCL day 7 samples have significantly more viable cells than day 1 samples. As a comparison between the E0 and E1 sub-groups, on day 7, a significant decrease in the number of cells in the E1 subgroup of PCL and CM-03K/PCL compared to E0 was observed. Phalloidin-DAPI staining also confirmed these results (Fig. 6).

**Fig. 7:**
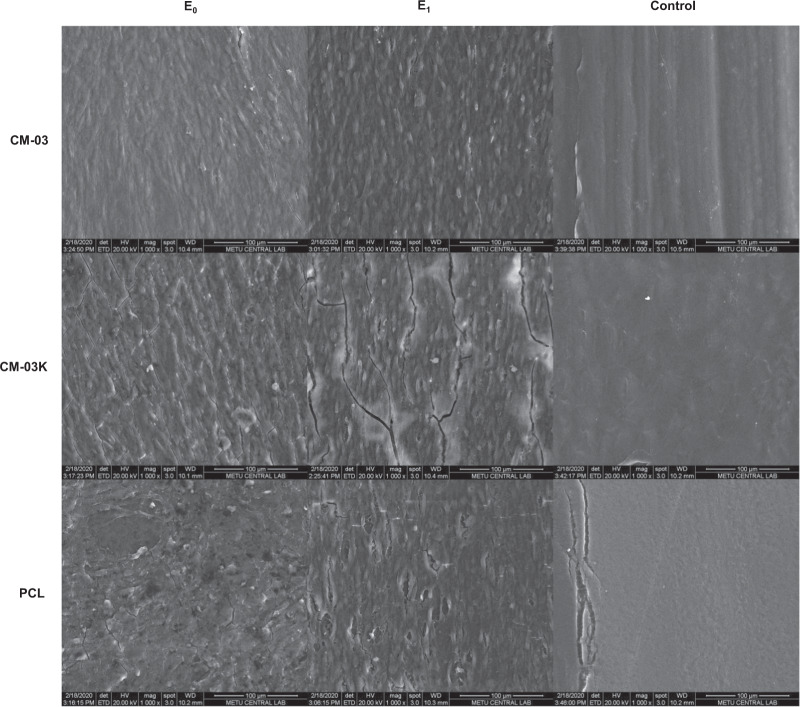
SEM micrographs of C2C12 cells on scaffolds without (E0) and with (E1) electrical stimulation on day 7. Scale bar 100 µm

SEM imaging revealed that cells attached and proliferated more readily on CM-03 scaffolds compared to PCL and CM-03K scaffolds both for the E0 and E1 sub-groups (Fig. 7). Cell viability of all groups was negatively affected to varying grades by electrical stimulation. It is known that electrical stimulation affects cell proliferation negatively [63]. Some studies also report that electrical stimulation can cause the formation of the pores in the cell membrane and thus may promote apoptosis [64–66]. While the metallic composition of the CMs is important to provide conductivity, the inorganic composition is also a critical parameter. CM-03 and CM-03K materials, synthesized by means of the green synthesis method, confirm this approach. CM-03K is more conductive than other samples due to its higher metal heavy content, on the other hand, after electrical stimulation, the inorganic composition of these materials may reduce number of viable cells on the surface. Unlike CM03-K, it has been evaluated that CM-03 is a more suitable material for cell culture in terms of both cell affinity and electrical conductivity due to its organic–inorganic structures [67].

### Immunostaining

**Fig. 8:**
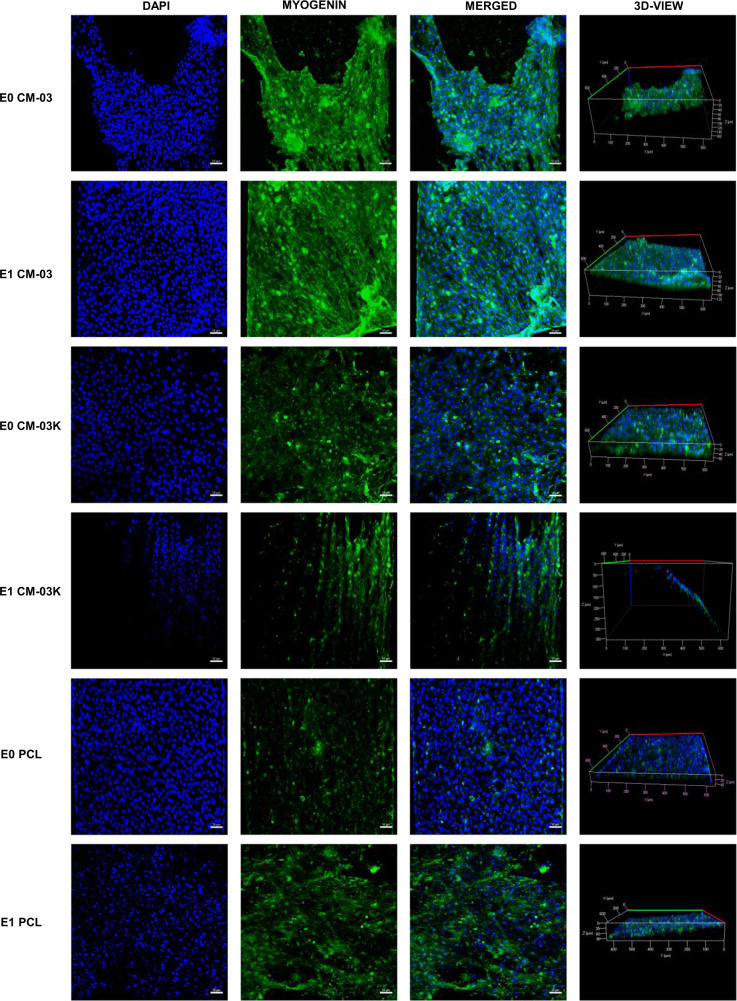
Immunofluorescent staining for myogenin (green). Cell nuclei stained with DAPI (blue). Scale bars 50 µm

**Fig. 9:**
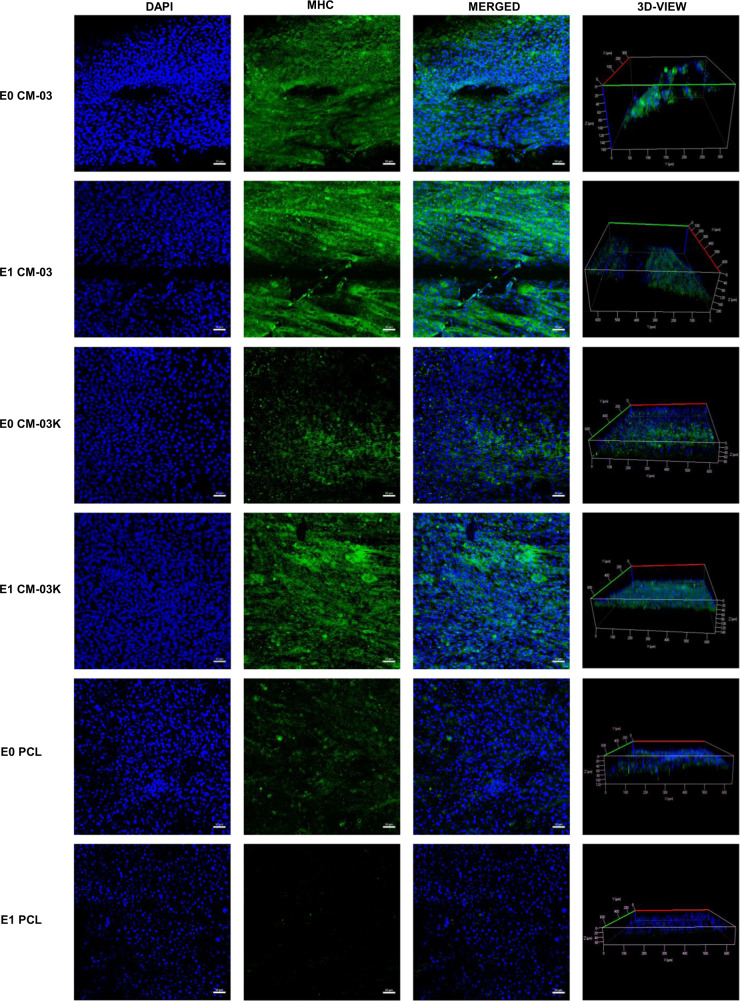
Immunofluorescent staining for myosin heavy chain (green). Cell nuclei stained with DAPI (blue). Scale bars 50 µm

**Fig. 10:**
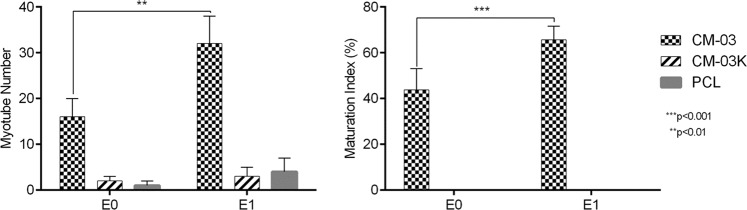
Quantification of the myotube number and maturation index calculated from MHC staining

In order to produce functional skeletal muscle tissue with tissue engineering technique, it is aimed to differentiate myoblasts into multinucleated myotubes. To examine the effect of electrical stimulation on myoblast differentiation, immunostaining was performed on CM-03 and CM-03K doped PCL scaffolds, and bare PCL as the control group. Immunofluorescent staining for myogenin (green) and MHC (green) was performed for all samples, and the corresponding images are presented in Fig. 8. On day 7, myogenin expression was observed for CM-03 samples for both E0 and E1 sub-groups. Within CM-03K and PCL E0 and E1 sub-groups, myogenin expression was observed weakly, while E1 sub-groups have more tubular geometry (Fig. 8). Myosin heavy chain was generally expressed during the muscle fiber formation [68] was detected strongly CM-03 E0 and E1 sub-groups (Fig. 9). According to the quantification analysis made using MHC fluorescent imaging, particularly in the E1 subgroup, the number of myotubes (**p < 0.01) and maturity of myotubes (***p < 0.001) was increased after electrical stimulation of the CM-03 significantly (Fig. 10). In other groups (CM-03K and PCL), fiber formation may have failed because insufficient cell proliferation could not support the formation of multinucleated cells under the electrical stimulation.

The results of the study represent that electrical cues may play a significant role in myotube formation. In addition, CM-03 doped scaffold and combining electrical stimulation indicated the highest differentiation of myoblasts. The CM-03 group that contains the metallic component as well as its organic and inorganic content, provides a more suitable extracellular matrix for use in skeletal muscle tissue engineering compared to the CM-03K group with includes only metallic content [69].

The adhesion and proliferation of the cells to the surface shows differences according to the chemical, physical and surface properties of the material [70, 71]. In addition, it is seen that the material has an inorganic and organic content that positively affects cell adhesion and differentiation [69, 72]. In this study, it has been shown that the conductive recycled carbonaceous material, CM-03 group, with its organic and inorganic content, constitutes a proper scaffolding material for cells to attach and proliferate for functional skeletal muscle tissue engineering approach.

The presence of metal ions including Al and Fe within scaffolds could constitute a problem in long-term applications considering their possible leach out from the scaffolds. This issue was also considered a problem for the use of metallic biomaterials since the corrosion and/or wear of these materials may lead to the release of metal ions which may lead to allergic and other undesired reactions intertwining the biocompatibility [73]. While this issue can be resolved by proper surface modification and passivation of metallic biomaterials [74], controlled release of metallic ions from scaffolds could also be used as a therapeutic strategy in tissue engineering [75]. Metallic ions such as Ca^2+^, Co^2+^, Cu^2+^, Ga^3+^, Fe^3+^, Mg^2+^, Ag^+^, Sr^2+^, Zn^2+^, and Mn^2+^ were incorporated within tissue engineering scaffolds and their controlled release were shown to have several positive effects including enhanced osteoconductivity and antibacterial efficiency [75]. On our study, the carbonaceous material has a metallic content including Al (1.5% w/w), Fe (1.4% w/w), Ca (19.3% w/w), and Si (6.1% w/w), from which 2% w/v were added into the scaffolds. Therefore, while the presence of the dilute concentrations of the metallic ions did not lead to the observation of any toxic effects in our system, in contrary, their presence enhanced the conductivity and lead to enhanced cellular activities. The relatively higher concentration of Ca (19.3% w/w) and Si (6.1% w/w) within the carbonaceous material also implies their potential use in bone tissue engineering applications.

## Conclusion

In this study, carbonaceous materials obtained from algae-based biomasses with natural conductivity using eco-friendly synthesis method were synthesized and successfully applied for the first time in the literature for the preparation of 3D printed electroactive scaffolds, as alternative materials to commonly used conductive materials (i.e., PANI, PPy, etc.) with various disadvantages. The critical effect of the second HTC was interpreted with the help of the XPS analysis technique. As well as its natural conductivity, the electrical conductivity of the carbonaceous material was improved by increasing the degree of graphitization (by the second HTC) and applying MWCNT catalysis. The electrical conductivity of carbon materials was supported by the interpretation of XRF and Raman spectroscopy analysis results. In addition, the results showed that CM-03 was more successful for providing cell proliferation due to its rich organic and inorganic content. The CM-03K sample showed more electrical conductivity due to its metal-heavy content. These promising results will fuel and guide the future studies to produce other novel carbon-based 3D printed scaffolds.
